# SMN Protein Can Be Reliably Measured in Whole Blood with an Electrochemiluminescence (ECL) Immunoassay: Implications for Clinical Trials

**DOI:** 10.1371/journal.pone.0150640

**Published:** 2016-03-08

**Authors:** Phillip Zaworski, Katharine M. von Herrmann, Shannon Taylor, Sara S. Sunshine, Kathleen McCarthy, Nicole Risher, Tara Newcomb, Marla Weetall, Thomas W. Prior, Kathryn J. Swoboda, Karen S. Chen, Sergey Paushkin

**Affiliations:** 1 PharmOptima, Portage, Michigan, United States of America; 2 Spinal Muscular Atrophy Foundation, New York, New York, United States of America; 3 PTC Therapeutics, South Plainfield, New Jersey, United States of America; 4 Department of Neurology, University of Utah School of Medicine, Salt Lake City, Utah, United States of America; 5 Department of Molecular Pathology, Wexner Medical Center, Ohio State University, Columbus, Ohio, United States of America; Iowa State University, UNITED STATES

## Abstract

Spinal muscular atrophy (SMA) is caused by defects in the survival motor neuron 1 (*SMN1*) gene that encodes survival motor neuron (SMN) protein. The majority of therapeutic approaches currently in clinical development for SMA aim to increase SMN protein expression and there is a need for sensitive methods able to quantify increases in SMN protein levels in accessible tissues. We have developed a sensitive electrochemiluminescence (ECL)-based immunoassay for measuring SMN protein in whole blood with a minimum volume requirement of 5μL. The SMN-ECL immunoassay enables accurate measurement of SMN in whole blood and other tissues. Using the assay, we measured SMN protein in whole blood from SMA patients and healthy controls and found that SMN protein levels were associated with *SMN2* copy number and were greater in SMA patients with 4 copies, relative to those with 2 and 3 copies. SMN protein levels did not vary significantly in healthy individuals over a four-week period and were not affected by circadian rhythms. Almost half of the SMN protein was found in platelets. We show that SMN protein levels in C/C-allele mice, which model a mild form of SMA, were high in neonatal stage, decreased in the first few weeks after birth, and then remained stable throughout the adult stage. Importantly, SMN protein levels in the CNS correlated with SMN levels measured in whole blood of the C/C-allele mice. These findings have implications for the measurement of SMN protein induction in whole blood in response to SMN-upregulating therapy.

## Introduction

Spinal Muscular Atrophy (SMA) is a genetically inherited neuromuscular disease, occurring in approximately 1 in 11,000 live births, and is the leading genetic cause of death in infants and toddlers (reviewed in [1]). Patients exhibit severe proximal muscle weakness and atrophy due low levels of survival motor neuron protein (SMN), which primarily results in degeneration of alpha-motor neurons of the anterior horn.

SMA presents as a continuous spectrum of symptoms that are clinically classified into four types of SMA dependent upon motor milestones achieved during development [2]. The most severe form of the disease, Werdnig-Hoffmann disease or Type I SMA, presents early in life; most infants are never able to sit independently and have a life expectancy of less than two years [3]. The intermediate form of the disease, Type II SMA (also known as Dubowitz disease), typically presents after 6 months of age. Type II patients achieve the ability to stay seated independently but are never able to walk independently. Survival rates for Type II were reported as 98.5% at 5 years and 68.5% at 25 years [4]. Type III SMA, also known as Kugelberg-Welander syndrome, is a milder form of the disease with symptoms typically presenting after children are already standing and walking. Life expectancy does not differ from the general population [4,5]. Type IV SMA has the mildest symptoms and presents in the second or third decade of life [6,7].

SMA is the result of a deletion or mutation in the survival motor neuron 1 (*SMN1*) gene that is located at 5q13.2 and encodes survival motor neuron protein (SMN). SMA patients have at least one copy of a paralogous gene, *SMN2*. *SMN*2 is almost identical to *SMN1*, but contains a C-T mutation that does not result in an amino acid change but affects the alternative splicing of *SMN*2 pre-mRNA so that exon 7 is frequently excluded from mRNA [8]. As a result, only a fraction (10–30%) of full length mRNA is produced, generating lower amounts of functional, full length SMN protein. The shortened mRNA, referred to as Δ7, encodes an unstable SMNΔ7 protein that is rapidly degraded [9–11]. Many of the current SMA drug development efforts target *SMN2* gene expression, aiming to modify *SMN2* splicing and increase the production of functional, full length SMN protein [12–14]. SMN is a ubiquitously expressed, intracellular protein known to be critically involved in snRNP assembly and the formation of other cellular RNPs containing coding and non-coding RNAs (reviewed in [15]). In addition, skeletal muscle fiber development and repair processes are sensitive to low levels of SMN protein [16–18]. Several groups are exploring a variety of approaches, including modulation of *SMN2* splicing, to upregulate expression of SMN protein; these efforts include a number of programs for novel antisense oligonucleotides, gene delivery vectors, and small molecules that are at various stages of preclinical and clinical development.

As therapeutics aimed at increasing SMN protein levels progress through clinical development, there is an increasing need for a pharmacodynamic marker to access target engagement and select the optimal dose for the therapeutics. To support successful clinical development of a treatment for SMA, it is important to be able to readily and accurately measure SMN protein in an accessible tissue, such as whole blood. Existing methods to measure SMN protein levels include an Elecsys platform-based assay developed by Roche Diagnostics to measure SMN in whole blood [19], an electrochemiluminescence immunoassay (ECLIA) to measure SMN in buccal cells [20], an SMN-ELISA developed to measure SMN in peripheral blood mononuclear cells [21], and western blotting and a homogeneous time-resolved fluorescence (HTRF) assay [12] to measure SMN protein in tissues homogenates. Here we report the development of a sensitive electrochemiluminescence (ECL)-based immunoassay for measuring SMN protein in as little as 5μL of whole blood. After validating the assay according to FDA guidelines, the assay was used to better understand SMN protein expression, stability and variability over time in whole blood of healthy individuals and SMA patients in non-interventional clinical studies at University of Utah and Jasper Clinic. These findings, in addition to the development of the SMN-ECL immunoassay capable of sensitively measuring SMN protein in whole blood, have a direct relevance for clinical development of SMA therapeutics.

## Materials and Methods

### SMN-ECL immunoassay protocol

The SMN-ECL immunoassay was developed in a sandwich immunoassay format. The calibrator for the assay was recombinant human SMN produced in *E*. *coli* (Enzo ADI-NBP-201). The mouse monoclonal capture anti-SMN antibody (clone 2B1, Enzo ADI-NBA-202, [22]) was solution coated onto Meso Scale Discovery (MSD) standard plates (30 μL/well) at a concentration of 1 μg/mL. After overnight incubation at 4°C, the plate was blocked with 5% BSA in PBS for 1 hour with shaking (650 rpm). Samples or standards were loaded at 25 μL per well. Sample buffer was composed of 50mM Tris, 500mM NaCl, 1% (w/v) BSA, 1% (v/v) Triton X-100, 0.05% (v/v) Protease Inhibitor Cocktail (Sigma, #P8340), pH 7.5. Samples and standards were incubated for 2 hours at room temperature with shaking (650 rpm). Primary and secondary detection antibodies were diluted into the following buffer: 50mM Tris, 137.5 mM NaCl, 1% (w/v) BSA, 0.05% (w/v) Tween 20, 0.2% mouse gamma globulin fraction (Rockland, #D-609-0200;), pH 7.5. Captured SMN protein was detected using a SULFO-TAG^™^ labeled rabbit polyclonal anti-SMN antibody (2 μg/mL, Protein Tech, Cat. No. 11708-1-AP). Samples were incubated with detection antibody for 1 hour with shaking at 650 rpm. Following incubation with the rabbit primary detection antibody and washing, samples were incubated with sulfotagged goat anti-rabbit antibody (0.5 μg/mL, MSD cat#R32AB-1) for signal amplification. Following incubation and washing, 150 μL/well 1X MSD read buffer T (Meso Scale Discovery R92TC-1) was added to each well and the plate was read using a Meso Scale Discovery Sector Imager 6000 instrument. SMN levels were determined from the standard curve via software provided with the Sector Imager 6000 instrument using a 4-parameter logistic fit equation.

### Antibody labeling

Rabbit anti-SMN antibody (Protein Tech, Cat. No. 11708-1-AP) was sulfotagged with caged ruthenium using 150 nmol sulfotag NHS ester and a 20:1 challenge ratio. The labeling reaction was performed using Meso Scale Discovery protocols (Meso Scale Discovery R91AN-1).

### Whole blood quality control preparation

Human whole blood was subjected to a freeze thaw event and was then assayed for SMN levels following a 1:40 dilution (the minimum required dilution, MRD). Prior to QC preparation, whole blood samples were assayed for endogenous levels of SMN. Three levels of QCs were prepared so that, after a 1:40 dilution, the following SMN levels would be achieved: 80% upper limit of quantitation (ULOQ, High), a mid-range sample (Mid), and 3X the lower limit of quantitation (LLOQ, Low). The High QC was generated by first adding 10X RIPA (Cell Signaling Tech #9806) buffer to the whole blood, resulting in a whole blood containing 1X RIPA. Following addition of RIPA buffer, the whole blood was spiked with purified SMN protein (Enzo) to a total SMN level (endogenous + spike) of 320 ng/mL. The Mid QC was undiluted whole blood. The Low QC was generated following dilution of whole blood to a level that would result in approximately 3X LLOQ following a 1:40 dilution into assay buffer.

### SMN ECL immunoassay qualification

The SMN ECL immunoassay was qualified for use in human whole blood using recommendations for the development of ligand binding assays found in Guidance for Industry Bioanalytical Method Validation, Draft Guidance published in 2013 [23]. Briefly the following studies were undertaken: selectivity, accuracy, precision, spike recovery, parallelism, reproducibility and stability. Stability studies included bench top stability, freeze thaw stability and long term storage stability.

### Whole blood fractionation

Human whole blood (WB) was fractionated into the following components: platelets, red blood cells, reticulocytes, peripheral blood mononuclear cells (PBMCs), and granulocytes. WB was collected into Citrate collection tubes. Platelets were isolated using slow speed centrifugation of WB. Tubes were centrifuged at 200 x g for 15 minutes at room temperature. The platelet rich plasma fraction (PRP, upper layer) was carefully removed from the tube and diluted 1:2 with Tyrodes buffer (5 mM HEPES, 134 mM NaCl, 12 mM Na-bicarbonate, 2.9 mM KCL, 0.34 mM Na-phosphate (dibasic), 1 mM MgCl_2_, 5 mM Dextrose, 1 μM PGE1 (prostaglandin E1), 3 mg/mL BSA, pH 7.4). Platelets were then pelleted by centrifugation at 800 x g and were frozen at -80°C until assay. PBMCs were isolated using density gradient centrifugation (Lymphoprep, Axis Shield cat# 1114544) and according to the manufacturer’s recommendations. Reticulocytes were separated from the WB fraction after removal of the PRP and PBMCs. Reticulocytes were isolated by immunoaffinity using anti-CD71 coated magnetic beads (Myltenii cat# 130-046-201) and manufacturer’s recommendation. Following removal of PRP and PBMCs, granulocytes were isolated by treating an aliquot of the red blood cell pellet with erythrocyte lysis buffer (EL buffer, Qiagen #1014614). After three rounds of EL buffer treatment and microscopic observation, the remaining cells were considered to be granulocytes.

### Study participants

Patients for this entire study were recruited at the University of Utah (Salt Lake City, Utah, US) under University of Utah Institution Review Board approved protocol 68133 (Swoboda). All procedures were conducted according to the principles described in the Declaration of Helsinki. Written informed consent was obtained from the subjects and their legal guardians as per institutional guidelines. All subjects had had prior clinical diagnostic testing confirming homozygous deletion of *SMN1*. Regardless of the availability of prior molecular diagnostic testing performed on a clinical basis, all samples were retested to confirm homozygous deletion of *SMN1* and determination of *SMN2* dosage in a single laboratory using previously published methods [24].

Healthy volunteers were randomly selected subjects donating blood at Jasper Clinic (Kalamazoo, MI). Venous and capillary blood was collected into K2-EDTA tubes using standard procedures. Samples were transported from the clinic to PharmOptima at ambient temperatures and then placed into a -80°C freezer. Total elapsed time between sample acquisition and freezing was less than 2 hours. All venous samples were aliquoted into 2 tubes prior to freezing. Capillary samples were frozen directly in the collection tubes.

### Cerebral spinal fluid (CSF)

Eleven human cerebral spinal fluid samples (Bioreclamation IVT) were evaluated for SMN protein and hemoglobin levels. Prior to SMN assay, samples were concentrated 10X using 10,000 MW cutoff spin filtration (Sartorius VS0101). Sample clarity and coloration, if any, were noted. Hemoglobin levels were assayed using a hemoglobin immunoassay (Bethyl Laboratories E88-135).

### Tissue sample preparation

Mouse tissue samples were maintained frozen at -80°C until homogenization. All tissues were homogenized in ER4 buffer (50 mM Tris, 300 mM NaCl, 10% (w/v) glycerol, 3 mM EDTA, 1 mM MgCl_2_, 20 mM β-glycerophosphate, 25 mM NaF, 1% Triton X-100) at a ratio of 10 μL/mg tissue. Tissues were homogenized using a Percellys homogenizer employing zirconium beads. Homogenates were clarified via centrifugation at 20,000 x g for 10 minutes at 4°C. Clarified supernatants were frozen and maintained at -80°C until the time of assay. Clarified homogenates were assayed for SMN at the following dilutions in assay buffer: brain (1:160), muscle (1:20) and spinal cord (1:80). SMN data was normalized to total soluble protein (ng SMN/mg protein). Total soluble protein was determined using the BCA protein assay (Pierce). The SMN-ELISA was conducted according to the manufacturer’s recommendations (Enzo ADI-900-209).

### SMA mice

Animal studies were done in an AAALAC-approved facility and approved by the Rutgers University Institutional Animal Care and Use Committee (IACUC). C/C-allele mice were kept in a controlled vivarium at ~25°C and ~50% humidity with a 12 h light/dark photoperiod and monitored for health. Mice had free access to food and water. The original breeding pairs of the C/C-allele mice (FVB.129(B6)-Smn1tm5(Smn1/SMN2)Mrph/J; stock number 008604) were obtained from The Jackson Laboratory (stock number 008604). This study was carried out in strict accordance with the recommendations in the Guide for the Care and Use of Laboratory Animals of the National Institutes of Health. The protocol was approved by the Committee on the Ethics of Animal Experiments of Rutgers (Permit Number: 000730). All efforts were made to minimize suffering. No animals were moribund or required euthanasia before the schedule necropsy. Animals were euthanized by carbon dioxide asphyxiation.

### Statistical Analysis

Variability in SMN protein levels over time in both human and mouse tissues was assessed by linear regression (p < 0.05). Correlation of SMN protein levels between tissues (venous blood versus capillary blood; whole blood versus spinal cord, brain, and muscle) was assessed using Pearson's correlation coefficient and linear regression for the specific two tissues being compared. Differences in SMN protein levels by *SMN2* copy number and SMA type were analyzed using one-way ANOVA and Tukey’s Multiple Comparison Test (p < 0.05). SMN levels in the spinal cord tissues of C/C-allele and WT mice were compared using an unpaired student’s t-test (p < 0.05), while SMN levels in whole blood of C/C-allele, heterozygous, and WT mice were assessed via one-way ANOVA and Tukey’s Multiple Comparison Test (p < 0.05). All statistical analyses were performed using GraphPad Prism 5.

## Results

### Development of an ECL immunoassay to measure SMN protein in whole blood

The SMN-ECL immunoassay is based on the Meso Scale Discovery platform, which enables sensitive detection of SMN protein. The assay was optimized to measure SMN protein in whole blood and validated according to FDA Guidelines [23]. Parallelism, selectivity, intra- and inter-run precision and accuracy were assessed (S1 Fig), and whole blood quality controls (QCs) were developed. At least three levels of QCs are included in every analysis of whole blood. The SMN-ECL assay has a dynamic range of 9.76 pg/mL to 20,000 pg/mL of SMN protein, exhibits no matrix effects, and requires as little as 5μL of whole blood for each analysis and a minimum dilution of 1:40 with the sample buffer. The lower limit of detection (LLOD) of the assay is 3 pg/mL.

### Guidelines for optimal whole blood handling conditions

**Fig 1:**
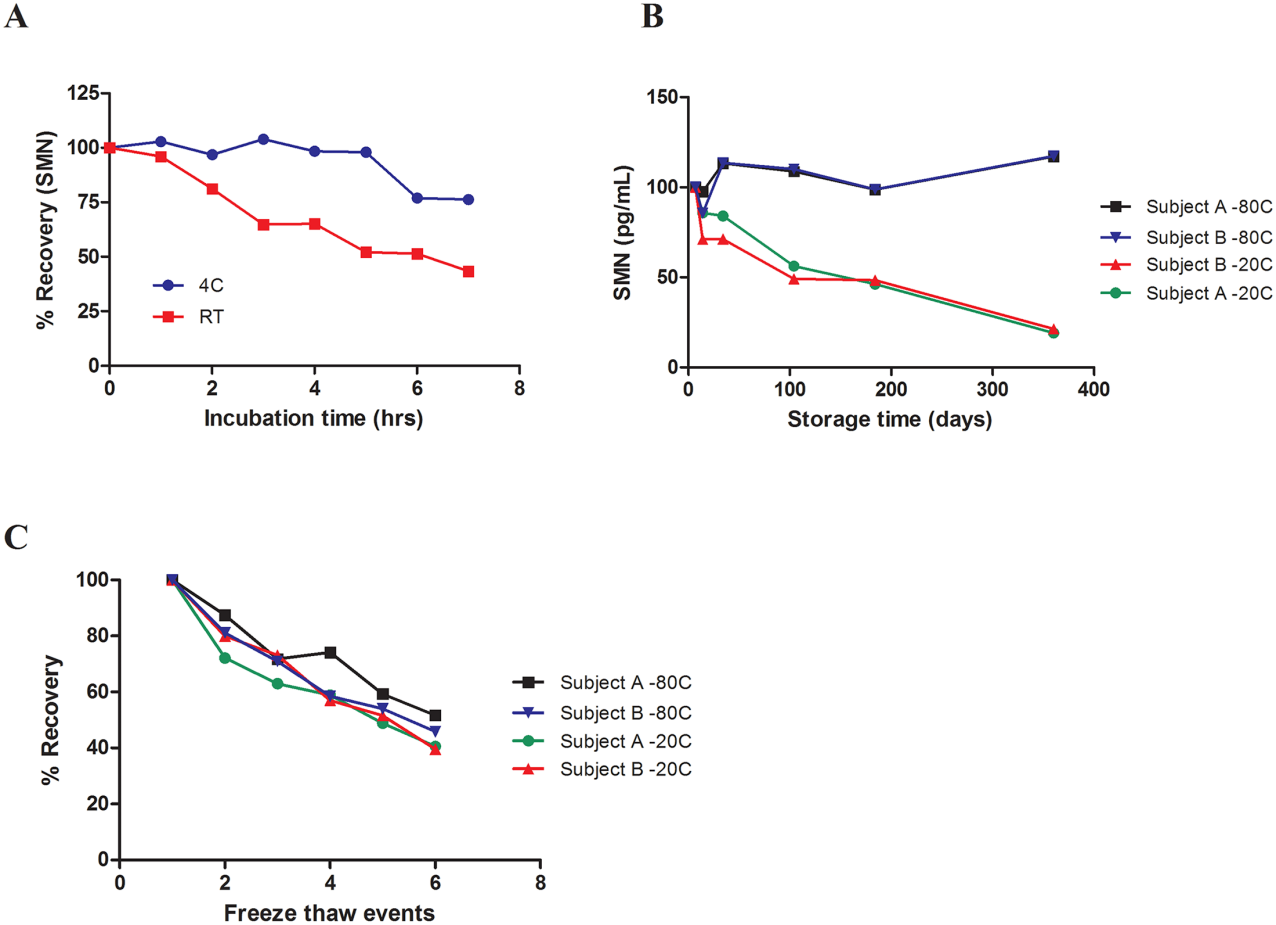
SMN protein stability in whole blood: short term, long term, and freeze / thaw events. Whole blood of healthy subjects was used in the study. (A) SMN protein was measured in previously frozen, undiluted whole blood samples incubated at 4°C or at room temperature. (B) SMN protein was measured in undiluted whole blood samples of two subjects stored at -80°C or at -20°C. (C) SMN protein levels were measured in samples of two subjects that went through freeze-thaw cycles. *FDA acceptance criteria (below 85%).

The SMN-ECL assay was used to characterize SMN protein stability in whole blood samples. We found that SMN protein in previously frozen, undiluted samples stored at -80°C was stable for up to 1 hour at room temperature and up to 5 hours at 4°C (Fig 1A). Previously undiluted frozen samples, thawed and diluted 1:40 with the sample buffer were stable at room temperature and at 4°C for up to 24 hours (data not shown). Long-term storage stability at -20°C and -80°C was assessed to provide guidance for whole blood sample storage. Prolonged incubation of whole blood at -20°C resulted in protein degradation. SMN levels in one whole blood sample stored at -20°C for 14 days fell below the 85% acceptance criterion set by the FDA Draft Guidance document; another sample showed a decrease in the SMN levels below 85% after one month of storage at -20°C (Fig 1B). SMN protein levels in both blood samples stored at -80°C remained stable throughout the course of the year-long study. To determine if SMN protein levels are affected by repeated freeze-thaw events, a separate set of the whole blood samples stored at -20°C and -80°C were repeatedly frozen, thawed, and assayed. Results from this experiment suggest that to prevent SMN protein degradation, whole blood samples should not be subjected to any freeze-thaw events regardless of storage temperature (Fig 1C).

### SMN protein expression levels do not vary substantially over time in circulating blood

**Fig 2:**
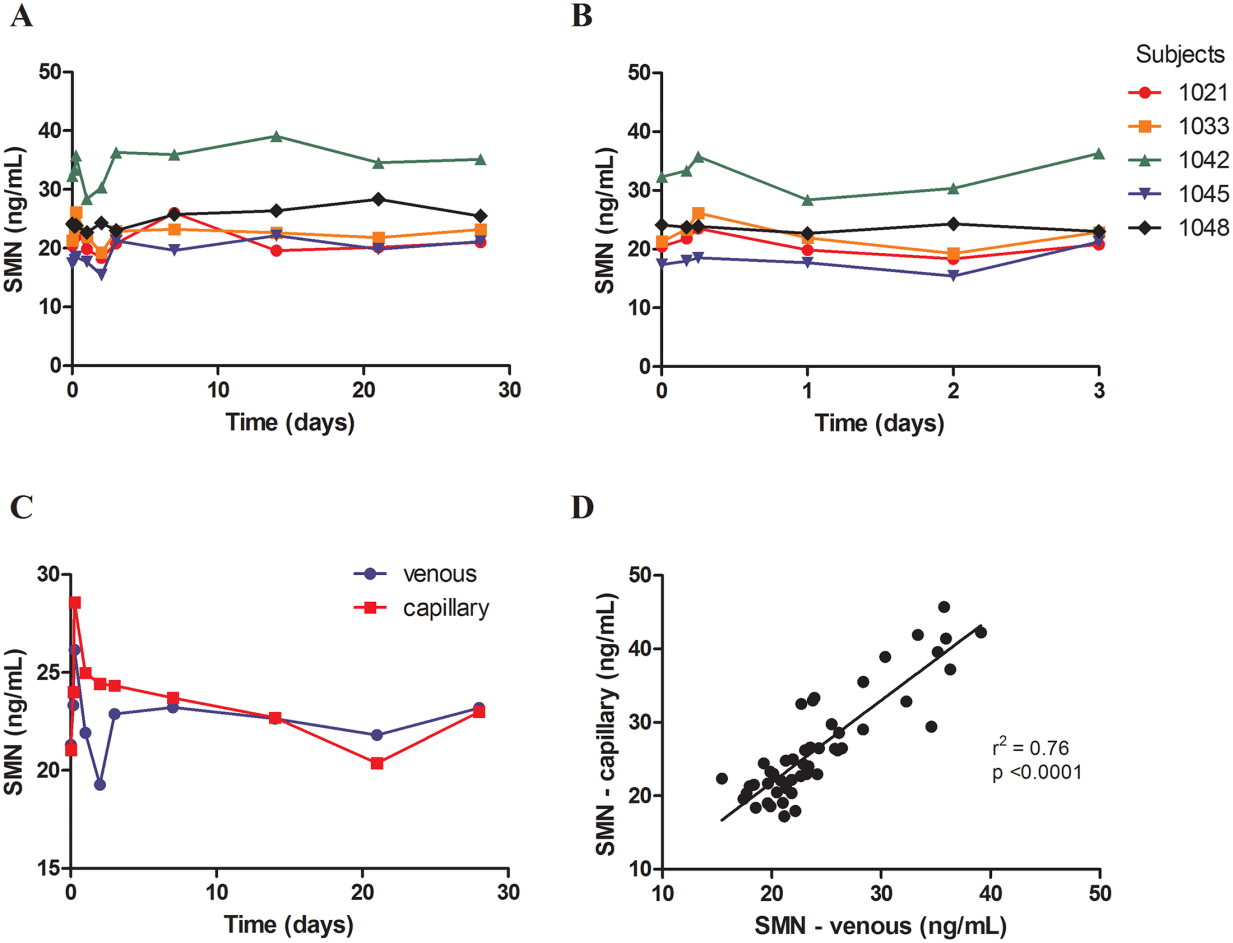
SMN protein levels in capillary and venous blood obtained over time from healthy volunteers did not vary significantly. Venous (A, B) and capillary (C) whole blood samples were obtained at 0, 4, 6, 24, 48, 72 hours and 1, 2, 3, 4 weeks from five healthy individuals. Fig 2B is an expanded version of Fig 2A. (D) SMN protein levels in capillary blood correlated significantly with SMN levels in venous blood (r^2^ = 0.76, p < 0.0001).

After assessing SMN protein stability in whole blood samples in response to laboratory manipulations, we set out to determine how levels of SMN protein in human whole blood change over the course of 24 hours and over several weeks. SMN protein was measured in whole blood collected at 0, 4, 6, 24, 48, 72 hours and 1, 2, 3, 4 weeks from healthy volunteers at the Jasper Clinic (MPI Research, Kalamazoo, MI). We found that SMN protein levels did not significantly vary over time and the standard deviations were around 10% of the mean (Fig 2A and 2B). Thus, any increase in SMN protein levels as a result of SMN-upregulating therapy should be at least 20% in order to be considered above the range of assay variability. Both venous and capillary whole blood samples were collected at each time point in all healthy volunteers to determine whether capillary blood collection could replace traditional, venous blood collection methods in clinical trials. SMN protein measured in capillary blood correlated 1:1 with SMN measures in venous blood (r^2^ = 0.76, p < 0.0001) and SMN protein variability over time was similar in both the capillary and venous blood (Fig 2C and 2D).

### The majority of SMN protein in whole blood is found in platelets

In order to determine the contribution of different cell populations to the SMN content of whole blood, we fractionated whole blood using methods designed to enrich for platelets, PBMCs, reticulocytes and red blood cells (RBCs). SMN protein was found in all cell fractions tested with the highest levels found in platelets (40–50% of total SMN measured in whole blood), followed by red blood cells/granulocytes (37–40%), PBMCs (18–20%) and reticulocytes (1–2%) (Table 1). The RBC and granulocyte fraction estimation was defined as the fraction not accounted for by the other cell types. In serum and plasma samples, SMN levels were below the level of detection, except in the case when there had been significant levels of hemolysis in the samples (data not shown).

**Table 1:** SMN protein was found in various cell types in whole blood. Platelets, red blood cells, PBMCs and reticulocytes were separated by a fractionation procedure.

Cell type	% total SMN in whole blood
Platelets	40.5 + 8.3
RBCs + Granulocytes*	45.8 + 7.4
PBMCs	19.4 + 2.6
Reticulocytes	1.9 + 0.6

*RBCs + Granulocyte fraction estimation was defined as the fraction not accounted for by the other cell types.

### SMN protein levels are significantly greater in SMA patients with 4 *SMN2* copies compared to patients with 2 and 3 copies of *SMN2*

**Fig 3:**
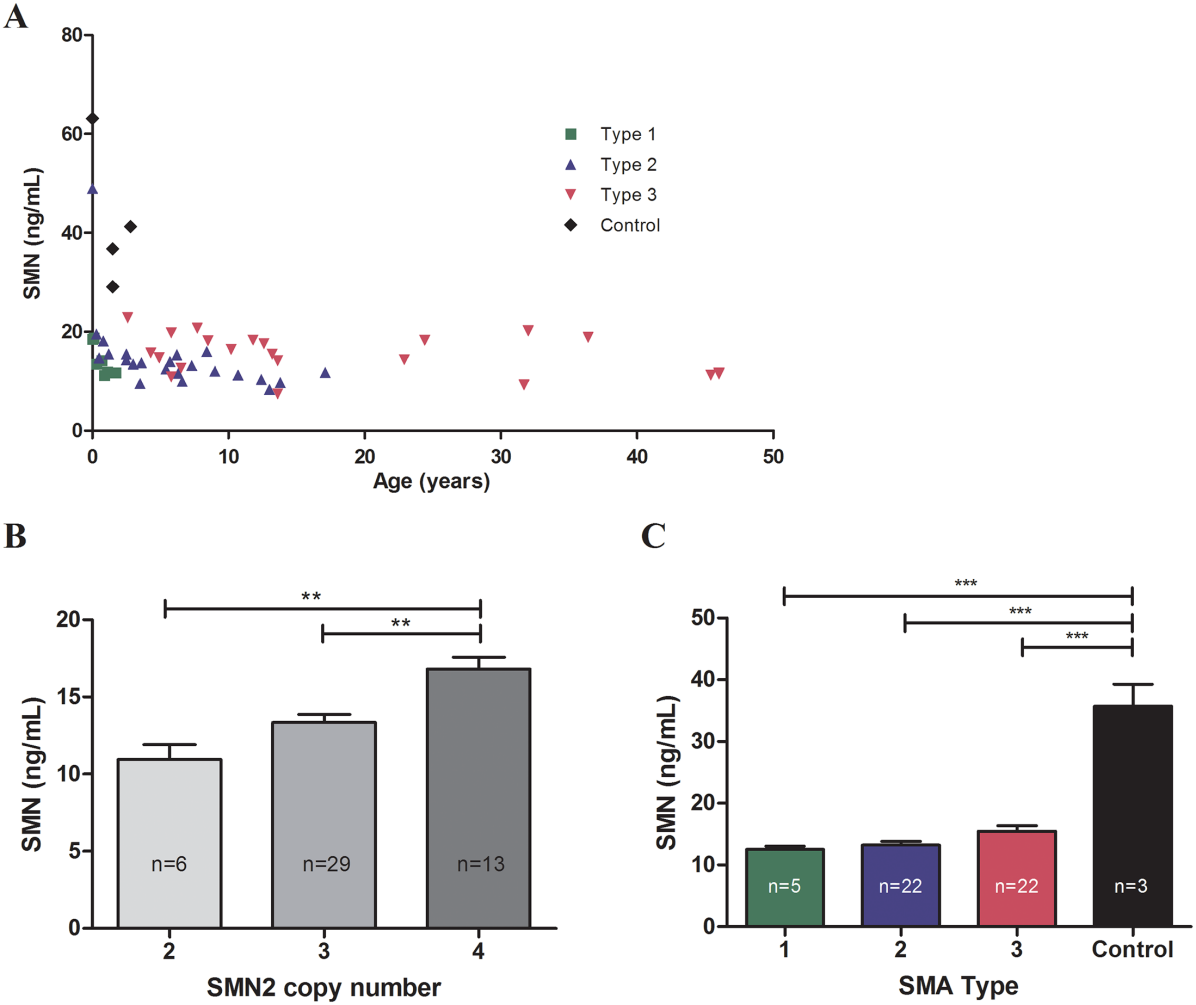
SMN protein levels in SMA patient and control whole blood samples. (A) SMN levels with respect to age in all subjects. (B) SMN protein levels were measured in SMA patients with 2, 3 and 4 copies of *SMN2*. In patients over 2 months of age, SMN levels were significantly greater in SMA patient samples with 4 *SMN2* copies relative to those with 2 and 3 *SMN2* copies (p = 0.0001). (C) SMN was also measured in three control samples and SMN levels were found to be significantly greater in the control samples relative to levels in SMA patients over 2 months of age (p < 0.0001).

The SMN-ECL assay was used to measure SMN protein in whole blood samples from SMA patients and healthy controls; protein levels were analyzed according to *SMN2* copy number, SMA type and age. Our sample set included patients with Type I, II and III SMA, ranging in age from 1 day to 46 years old. Samples from four healthy controls under 3 years of age were included in the analysis. Within this data set, we did not observe a significant change in SMN protein levels with increasing age in individuals older than two months (Fig 3A). There may be a decline in the SMN levels in the first months after birth but more data from that age group are needed to confirm this trend. Our sample set included only three patients younger than 2 months of age and while one of these patients exhibited higher SMN levels than older individuals, the low sample number precludes us from making a definitive conclusion regarding SMN protein levels in newborns. When patients younger than 2 months were excluded due to variability in this age group, SMN protein levels were found to be associated with *SMN2* copy number and were found to be significantly greater in SMA patients with 4 copies of *SMN2*, relative to patients with 2 and 3 copies of the gene (p < 0.0001) (Fig 3B). This association was not seen when all patients were included in the analysis. In both analyses, there was no significant difference in SMN levels between patients with 2 and 3 copies of *SMN*2. When separated by SMA Type, SMN protein levels measured in the healthy controls were significantly higher than levels in SMA Type I, II and III patients (p < 0.0001) (Fig 3C). However, we did not find a significant difference in SMN protein levels between SMA types.

### SMN protein is not detectable in CSF samples

While the SMN-ECL immunoassay was developed with the intent to quantify SMN protein in whole blood, the assay is also capable of sensitively measuring SMN protein in other human tissues as well as in murine tissues. As there are some SMA therapies being developed that utilize intrathecal injection, SMN levels in human cerebral spinal fluid (CSF) could be an important biomarker, but there is limited information about whether SMN is present in this tissue. For this reason, we evaluated human CSF samples for SMN protein levels. To control for possible blood contamination of CSF samples, which could contribute to SMN levels, CSF samples were also monitored for hemoglobin, a protein normally not found in CSF. CSF samples from 11 healthy controls were concentrated 10 fold (increasing the effective sensitivity of the SMN-ECL assay from 3 pg/mL to 0.3 pg/mL) and concentrates were assayed for both SMN protein and hemoglobin. Results indicate that SMN protein in CSF was detected only in samples that also had detectable levels of hemoglobin, suggesting that the presence of SMN may be due to contamination of the CSF samples with whole blood (Table 2). Measurement of SMN protein in CSF should thus be interpreted cautiously due to possible contamination with blood.

**Table 2:** SMN only detected in cerebral spinal fluid samples containing hemoglobin. The CSF samples obtained from healthy volunteers were concentrated prior to analysis, and the sensitivity of the SMN-ECL immunoassay was 0.3 pg/mL. Hemoglobin was measured using a hemoglobin immunoassay (Bethyl Laboratories E88-135). Approximately 3 pg of SMN correspond to 10,000 ng of hemoglobin in 1 mL of whole blood from a healthy adult. LLQ: lower limit of quantification.

Sample	SMN (pg/mL) CSF supernatant	SMN (pg/mL) CSF pellet	Hemoglobin (ng/mL)
1	LLQ	LLQ	11
2	2.0	LLQ	5,686
3	LLQ	LLQ	LLQ
4	2.7	LLQ	1,176
5	2.8	LLQ	6,486
6	LLQ	LLQ	12
7	LLQ	LLQ	18
8	LLQ	LLQ	164
9	LLQ	LLQ	750
10	LLQ	LLQ	2,501
11	34.2	0.8	55,311

### SMN-ECL can be used to measure SMN protein in other tissues and species

**Fig 4:**
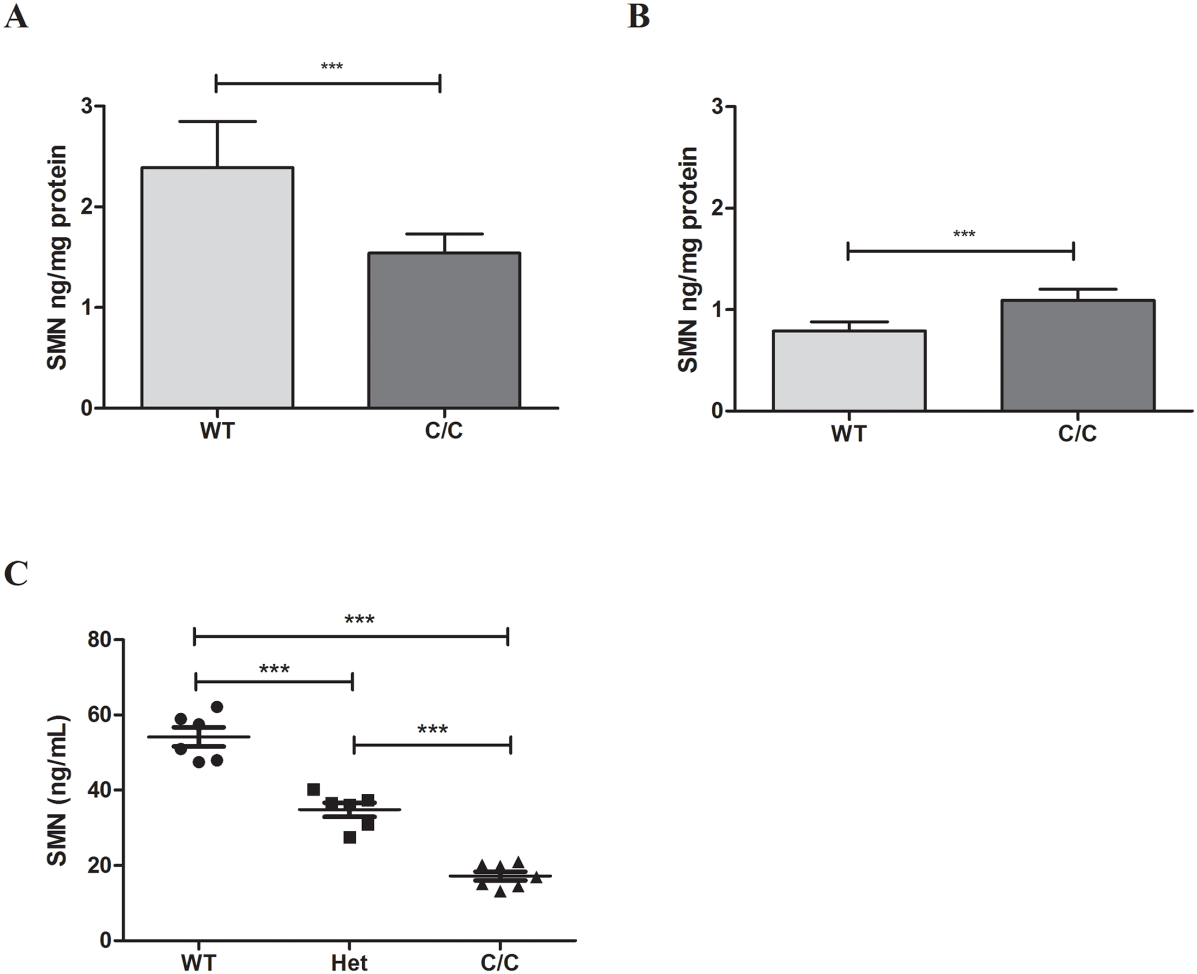
SMN protein levels in tissues of C/C-allele and WT mice measured by SMN-ECL and SMN-ELISA. Protein levels were measured in the spinal cord of C/C-allele and WT mice using (A) SMN-ECL and (B) SMN-ELISA. Both assays showed a statistically significant difference in SMN levels between WT and C/C-allele mice (p < 0.0001). (C) SMN protein levels in the whole blood of C/C-allele, WT and heterozygous mice measured by SMN-ECL.

In addition to measuring SMN protein in human tissues, the SMN-ECL immunoassay is able to sensitively measure SMN protein in murine tissues including whole blood. This is notable because SMN levels in whole blood cannot be assayed with the SMN-ELISA due to matrix effects. In addition to being able to measure SMN in whole blood, the SMN-ECL assay has been used to measure SMN in mouse quadriceps, heart, liver, brain, whole blood and spinal cord tissues in various mouse models. When we compare the SMN-ECL assay to the commercially available SMN-ELISA [21], our results indicate that the ECL-based method is more sensitive and provides more accurate results in tissue homogenates (data not shown). In this study, we used the C/C-allele SMA model that represents a mild form of the disease. These mice have normal life expectancy but they exhibit muscle weakness, peripheral necrosis, and reduced body weight [25]. While both SMN-ECL and SMN-ELISA assays show a statistically significant difference in SMN levels in tissues between wild type (WT) and C/C-allele SMA mice, the SMN-ECL assay correctly measured lower SMN levels in the C/C-allele mice relative to those in WT mice whereas the ELISA detected higher SMN protein in the C/C-allele mice relative to those in the WT mice (Fig 4A and 4B). This outcome may be caused by matrix effects in SMN-ELISA or higher affinity of SMN-ELISA toward murine Smn protein vs. human SMN protein. The SMN-ECL assay was able to reliably differentiate between WT, heterozygote, and C/C-allele mice (Fig 4C).

### SMN protein levels rapidly decrease during early development in mice

**Fig 5:**
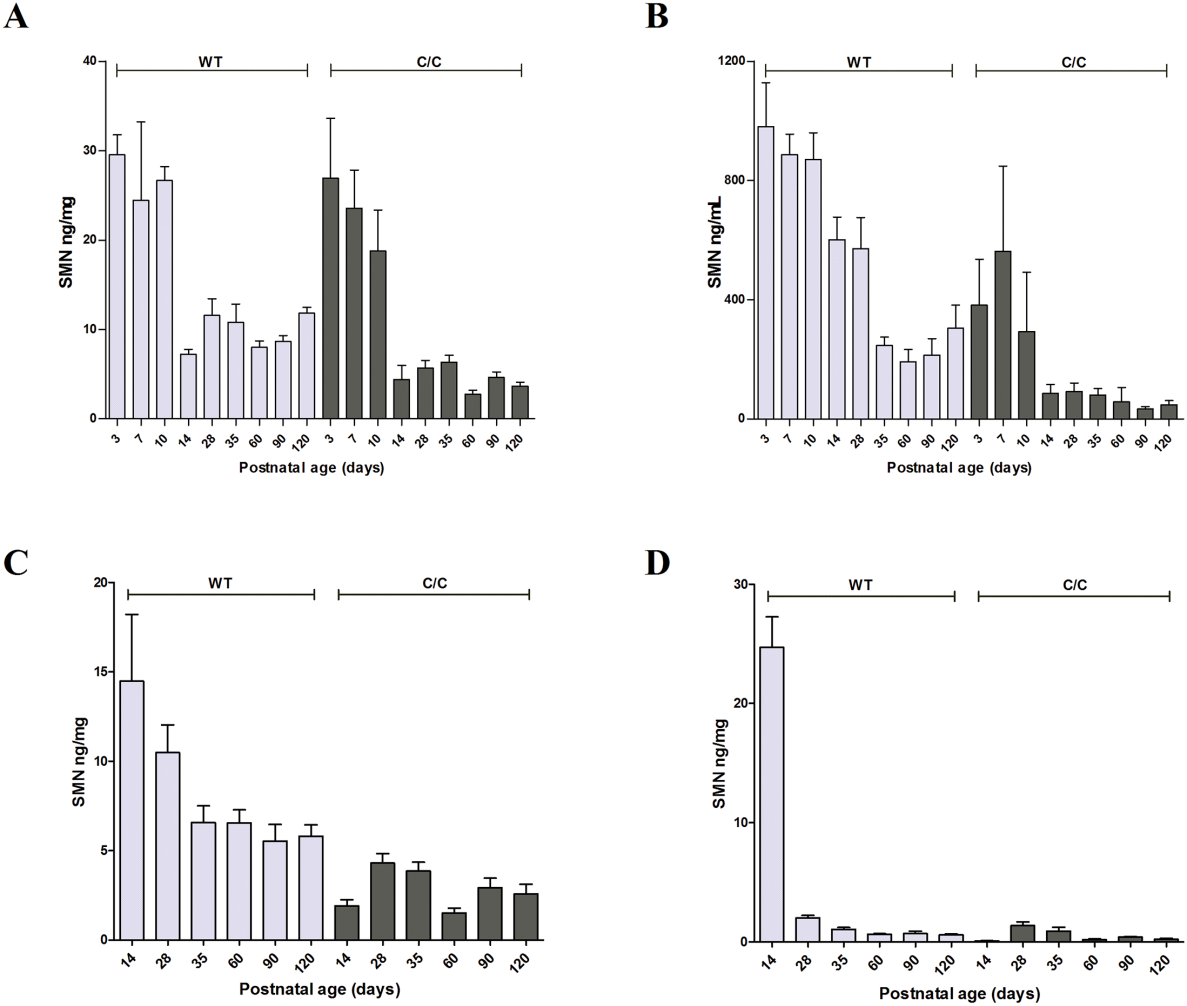
SMN protein levels in WT and C/C-allele SMA mice of different ages. SMN protein levels were measured by SMN-ECL at various days post-birth in (A) brain, (B) whole blood, (C) spinal cord, and (D) gastrocnemius muscle.

To assess SMN protein expression levels during development, we used the SMN-ECL to measure SMN in a variety of tissues that were harvested from C/C-allele mice and WT mice at various time points between birth and 120 days post-birth (spinal cord and gastrocnemius muscle tissues were collected on postnatal days 14, 28, 35, 60, 90, and 120; whole blood and brain tissues were also collected on postnatal days 3. 7, and 10 in addition to the aforementioned time points). In brain, SMN protein levels decreased during the first two weeks of life and remained steady afterwards for both WT and C/C-allele mice (Fig 5A). Similarly, SMN protein levels in whole blood of C/C-allele mice declined until PND14, at which point SMN protein levels stabilized (Fig 5B). However, in WT mice this stabilization occurred only after PND35. Spinal cord and muscle tissues were not collected before PND14 in this study, but the data for these tissues also indicate a similar trend wherein SMN protein levels are elevated at PND14 and then decline (Fig 5C and 5D). In spinal cord, SMN protein was stable from PND14 onward in C/C-allele mice but continued to decline until PND35 in WT mice (Fig 5C), similarly to what was found in whole blood. In muscle, in WT mice SMN protein levels were remarkably high at PND14, but decreased at PND28 and PND35 before stabilizing (Fig 5D). Interestingly, in C/C-allele mice, muscle tissue SMN levels were low at day 3 and remained low during the entire time course evaluated. In a separate study where spinal cord and muscle tissue were collected on days 3, 9, 14, 42, 84, 175, 225, and 294 of life, levels of SMN protein were found to decrease in the first two-three weeks of life (data not shown).

**Fig 6:**
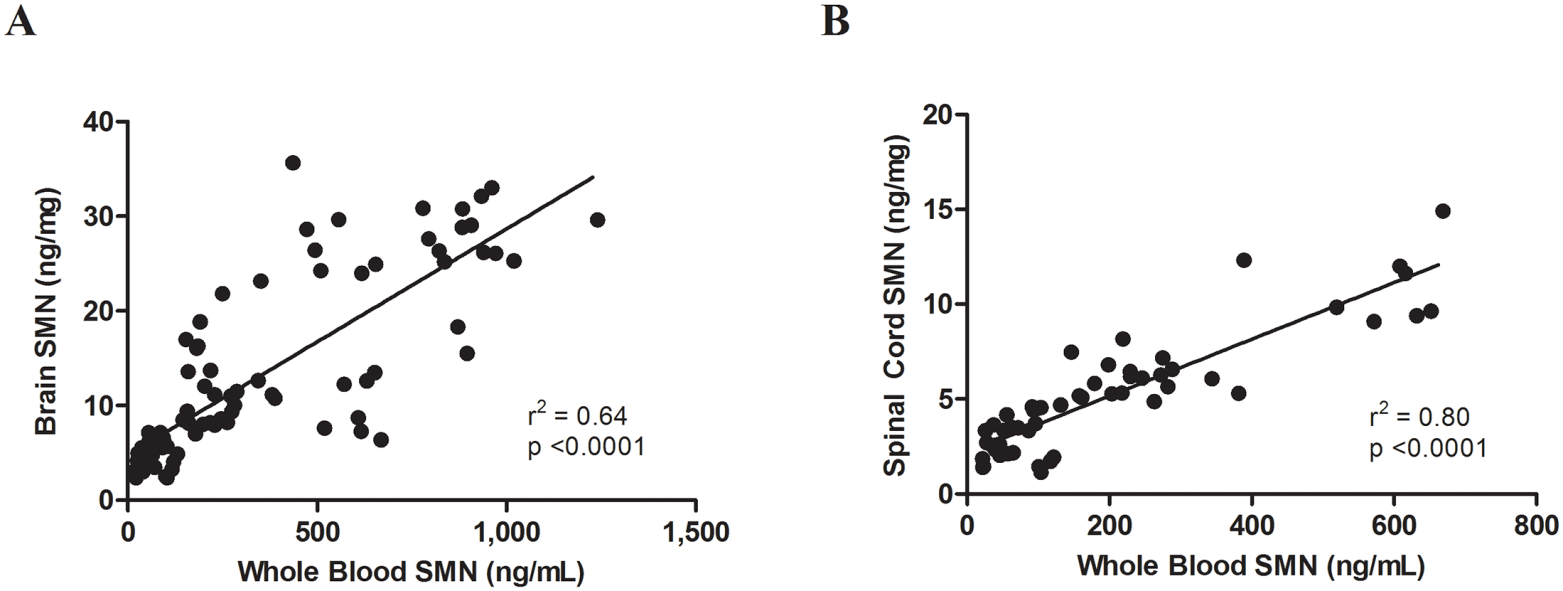
Correlation of SMN protein levels between tissues. SMN protein levels were correlated in WT and C/C-allele SMA mice between (A) whole blood and brain and (B) whole blood and spinal cord.

Absolute SMN protein levels in WT mice were the highest in the brain, followed by those in spinal cord and then muscle. For both WT and C/C-allele mice, SMN protein levels in whole blood correlated well with SMN levels in brain (p = < 0.0001 and r^2^ = 0.64) (Fig 6A) and spinal cord (p = < 0.0001 and r^2^ = 0.80) (Fig 6B). This correlation supports the use of SMN measurements of whole blood as a biomarker for evaluating CNS SMN protein in clinical trials.

## Discussion

We have developed a sensitive assay to measure SMN protein in whole blood and other tissues. The assay has been validated for use in clinical trials according to FDA guidelines [23] and can be utilized to measure SMN protein in clinical trials in SMA. We report here that SMN protein expression in healthy adults does not vary significantly over time and is not affected by circadian rhythms. We found that both capillary and venous whole blood can be used to measure SMN protein levels reliably; however, we recommend using a consistent method of whole blood collection within a clinical trial. In addition to measuring SMN protein in human whole blood and CSF, the assay has been used to measure SMN protein levels in brain, spinal cord, and iliopsoas muscle tissues obtained from SMA patient autopsy materials (courtesy of Dr. Charlotte Sumner, personal communications). The assay can also detect and sensitively measure SMN protein in mouse tissues, a capability that has been used to characterize SMN protein expression during development and that can be used to accelerate preclinical development of SMA therapeutics. This newly developed, validated SMN-ECL assay has the ability to positively impact translational and clinical research, significantly contributing to clinical development of SMA therapeutics.

A similar assay (ECLIA) based on MSD technology was recently published. The assay, sensitivity of which is 10-fold less compared to the SMN-ECL, was used for the measurement of SMN protein in buccal cells obtained from four healthy controls, one SMA carrier, and one SMA patient [20]. The SMA patient sample had approximately twice as much SMN protein as was measured in the control samples. This result differs significantly from the findings reported by the present study and by a recently published study [26], measuring SMN protein in whole blood samples and PBMCs respectively, suggesting that buccal cells may not be the most reliable cell type to detect SMN protein for use as a biomarker in clinical development of SMA therapeutics.

SMN protein has been previously measured in PBMCs isolated from whole blood using a commercially available ELISA (ADI-900-209, EnzoLifeSciences) [21]. While specific for SMN protein, the SMN-ELISA is not suitable for measuring SMN protein in whole blood. As we report in this study, PBMCs contain only 20% of the total SMN protein in blood and thus may not provide an accurate representation of the total SMN protein in whole blood. In addition, it was found that SMN levels fluctuate in PBMCs due to activation of the cells as a result of an inflammatory response [27]. In this study, we used both the SMN-ECL assay and SMN-ELISA to measure SMN protein in spinal cord samples obtained from WT and C/C-allele mice. SMN protein levels in C/C-allele mice were lower compared to WT mice when measured with the SMN-ECL assay, however the SMN-ELISA showed more SMN in C/C-allele mice relative to WT mice. The ability of SMN-ECL to correctly differentiate between the SMA and healthy mice could be due to the different assay platforms and/or buffer conditions.

Our results regarding the correlation between SMN protein levels and SMA type and *SMN2* copy number are consistent with previously published data from the BforSMA study [26]. In both studies, SMN protein levels were significantly greater from healthy volunteers relative to those in samples from patients with all types of SMA. There were no significant differences in SMN protein levels between SMA types. These results are also consistent with recently published work that shows a significant overlap in SMN protein levels between SMA patients of various types [19]. While a correlation between SMA disease severity and SMN protein levels has been previously reported, the increased sensitivity and greater sample size of recent studies have shed light on the initial findings [26,28]. The significant overlap in SMN protein levels between SMA types reported in the present study suggests that a small increase in SMN protein levels could result in significant phenotypic changes, which was also supported by the finding that a single base substitution in exon 7 (859G>C) positively influences disease severity despite only increasing exon 7 inclusion by 20% [29]. We also confirmed that when all patients were grouped together, *SMN2* copy number significantly correlated with SMN protein levels (r^2^ = 0.48, p = 0.0005). In addition, SMA patients older than two months with 4 copies of *SMN2* had significantly higher levels of SMN protein relative to patients with 2 or 3 copies of *SMN2*. Another group measured SMN protein levels in PBMCs using the SMN-ELISA and found no correlation between SMN protein and either *SMN2* copy number or clinical phenotype in a cohort of adult, SMA Type IIIa and IIIb patients [30]. The increased sensitivity of the SMN-ECL assay, 3 pg of SMN per 1 mL of whole blood, relative to the sensitivity of the SMN-ELISA reported to be 50 pg/mL [21], and the ability to sample the whole blood instead of just the PBMCs likely contribute to the ability to see a statistically significant difference in SMN protein levels between 4 *SMN2* copies and both 3 and 2 *SMN2* copies in the present study.

While the majority of SMN protein was found to be in the platelet and red blood cell fractions, a significant amount of protein (20%) came from lymphocytes suggesting that lymphocytes, which comprise less than 1% of the total number of cells in whole blood, are highly enriched with SMN protein. Platelets were found to contain between 40–50% of the total SMN protein measured in whole blood, and to be more highly enriched with SMN relative to red blood cells. We were surprised to find such a significant percentage of the entire measurable fraction of SMN protein in platelets, raising the question of why SMN protein is so abundant in a population of cells that comprises such a small fraction of whole blood (4–7% of all cells). It has been previously reported that mature platelets contain many proteins involved in spliceosome activity, including SMN protein [31]. Platelets are produced by megakaryocytes and while they do not contain nuclei, platelets do harbor pre-mRNA that requires processing before translation into protein. It has been shown that interleukin-1B is alternatively spliced in platelets [31], but it is unknown whether they can generate *SMN* transcripts. The SMN protein detected may have a variety of functions in platelets in addition to involvement in splicing activity, and/or megakaryoctyes may be highly enriched with SMN protein resulting in the formation of platelets with significant levels of SMN protein. Further analysis of the function of SMN protein in each cell type in whole blood may unveil new aspects of its function.

Findings from the fractionation experiment have important implications for measuring SMN protein in whole blood after treatment with SMN-upregulating therapeutics. Platelets, lymphocytes, and red blood cells turn over at different rates (approximately 10, 16 and 150 days respectively), which will impact measureable responses to drug treatments aimed at increasing SMN protein levels. Lymphocytes are the only cell types in whole blood that can definitively process pre-mRNA and produce proteins. There is limited information about splicing capability of granulocytes. Systemic administration of small molecules that modulate alternative splicing will quickly result in the increase of *SMN2* full length mRNA and subsequent increase in SMN protein in lymphocytes and possibly granulocytes. However, small molecule splicing modulators should not affect SMN levels in platelets and RBC circulating in whole blood, as these cells do not process pre-mRNA. SMN protein levels will only change in newly generated RBC and platelets that are produced in bone marrow while in the presence of splicing modulators. Therefore, in order to see a full response of the drug on the SMN protein levels in the whole blood, it will be necessary to wait until platelets and RBCs, the cells that contribute the majority of the SMN protein to whole blood, have turned over and been replaced with new cells generated in the presence of drug. Rough calculations suggest that one should expect to see substantial changes in SMN protein levels after two weeks of treatment, although it could take much longer to see the maximal effect. Because of this, it is important to consider the timing of SMN measurement and how much change should be expected when designing clinical trials.

Finally, we measured SMN protein levels over time in a variety of tissues from a mild mouse model of SMA (C/C-allele) to determine expression patterns during development that may be used to predict SMN expression time course in humans. In all C/C-allele mouse tissues assessed, SMN protein levels decreased in the first few weeks after birth and then remained stable throughout the course of the study. These results are consistent with the SMN protein decrease observed between PND3 and PND14 in tissues of delta7 SMA mice [21]. A recent publication from the NeuroNEXT SMA biomarker study found that SMN protein levels in PBMCs did not correlate with age in healthy infants (under 26 weeks of age), but did decline in SMA patients of the same age range [32]. SMN protein has been previously reported to play a significant role in development of motor neurons, neuromuscular junctions, and skeletal muscle, processes that are very active during the beginning of life [33]. In addition to decreasing in the first 21 days after birth in C/C-allele mice, SMN protein levels measured in brain, spinal cord, and whole blood were found to correlate significantly between tissue types. This finding validates the use of SMN protein in whole blood as a biomarker, as it increases confidence that changes observed in an accessible, tissue in the periphery—whole blood—would provide an accurate representation of changes in SMN protein levels in the central nervous system.

As therapeutic treatments continue to progress through clinical development, there is a need not only to accurately measure SMN protein but also to characterize SMN protein expression, stability, and variability over time. We have developed a sensitive assay to measure SMN protein in whole blood, and have demonstrated that SMN protein remains stable when stored at -80°C, does not significantly fluctuate over time in circulation, and correlates with *SMN2* copy number in SMA patients. These results, among others presented in the study, aim to support clinical development of SMN-upregulating therapeutics for the treatment of SMA.

## Supporting Information

S1 Fig Parallelism and selectivity results from the feasibility study of the SMN-ECL assay qualification. (A) The parallel nature of the curves indicates the absence of matrix effects. (B) Spike recovery was conducted with 6 whole blood matrices to assess selectivity, matrix one shown, and results were within the FDA acceptance criteria (80–120%). *1:40 was chosen as the minimum required dilution (MRD) because it was in the middle of the standard curve and exhibited no matrix effects. For the purposes of demonstrating parallelism the graphs for whole blood +/- spike were offset from the SMN calibration curve.
(TIF)
