# Supplementary figures and images for: SMN Protein Can Be Reliably Measured in Whole Blood with an Electrochemiluminescence (ECL) Immunoassay: Implications for Clinical Trials
Source: PLoS One. 2016 Mar 8;11(3):e0150640. doi: 10.1371/journal.pone.0150640 (PMC4783032; doi:10.1371/journal.pone.0150640)

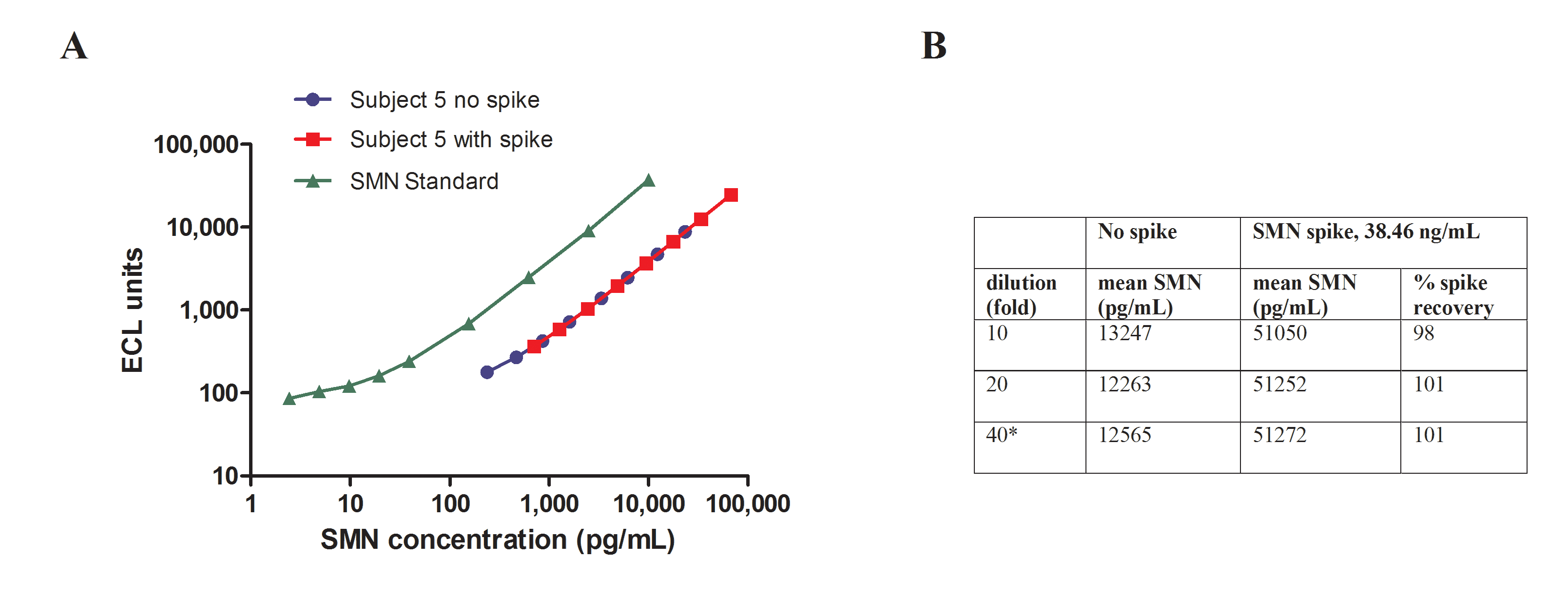

Supplement: S1 Fig — (A) The parallel nature of the curves indicates the absence of matrix effects. (B) Spike recovery was conducted with 6 whole blood matrices to assess selectivity, matrix one shown, and results were within the FDA acceptance criteria (80–120%). *1:40 was chosen as the minimum required dilution (MRD) because it was in the middle of the standard curve and exhibited no matrix effects. For the purposes of demonstrating parallelism the graphs for whole blood +/- spike were offset from the SMN calibration curve. (TIF) [file pone.0150640.s001.tif]
